# Clinical and behavioural features of *SYNGAP1*-related intellectual disability: a parent and caregiver description

**DOI:** 10.1186/s11689-022-09437-x

**Published:** 2022-06-02

**Authors:** Damien Wright, Aisling Kenny, Sarah Eley, Andrew G. McKechanie, Andrew C. Stanfield

**Affiliations:** Patrick Wild Centre, Division of Psychiatry, Kennedy Tower, Royal Edinburgh Hospital, University of Edinburgh, Edinburgh, EH10 5HF Scotland

**Keywords:** *SYNGAP1-*related ID, Behavioural phenotype, Intellectual disability, Autism

## Abstract

**Background:**

*SYNGAP1*-related intellectual disability (ID) is a recently described neurodevelopmental disorder that is caused by pathogenic variation in the *SYNGAP1* gene. To date, the behavioural characteristics of this disorder have mainly been highlighted via the prevalence of existing diagnoses in case series. We set out to detail the behavioural features of this disorder by undertaking interviews with those who have a child with *SYNGAP1*-related ID to allow them to describe their child’s behaviour.

**Methods:**

We conducted 27 semi-structured interviews with parents and caregivers which covered basic information (e.g., age, gender), family history, perinatal history, past medical history, developmental history, epilepsy, behavioural history, and a general description of their child’s behaviour.

**Results:**

Using a mixed quantitative and qualitative approach, the responses from the parents indicated that those with *SYNGAP1-*related ID showed high rates of autism spectrum disorder (52%), difficulties with fine and gross motor skills, delays in language development, and a high prevalence of epilepsy (70%). A qualitative analysis highlighted their general behaviour affected the themes of daily living skills, distress-related behaviours, emotional regulation, difficulties with change, a lack of danger awareness, and sensory differences. Sensory features described involved auditory, visual, tactile, gustatory, and proprioceptive themes.

**Conclusions:**

Our findings and behavioural descriptions provide important insights as well as implications for the diagnosis and care of those with *SYNGAP1-*related ID.

**Supplementary Information:**

The online version contains supplementary material available at 10.1186/s11689-022-09437-x.

## Background

Neurodevelopmental disorders are conditions in which the brain develops differently than is typically expected and as a result leads to functional impairment. These disorders are typically evident in early life and can affect a wide variety of domains including learning, memory, motor skills, communication, and emotions. One neurodevelopmental disorder, intellectual disability (ID), is characterised as significant global cognitive impairment (specifically an IQ < 70) and difficulties in adaptive functioning which became apparent before the age of 18 and is estimated to affect around 2% of the population [16, 22]. Individuals with ID are recognised to be amongst the most disadvantaged social groups [2, 12, 17, 26].

One of the most recognised causes of sporadic ID (i.e., non-inherited) is mutation of the *SYNGAP1* gene, which causes *SYNGAP1-*related ID, and is said to account for between 0.5–1% of all cases [3, 8, 10, 11, 25]. The *SYNGAP1* gene encodes for the ras-GTPase activating protein, SYNGAP1, which plays an essential role in brain function and development. De novo *SYNGAP1* gene mutations were first reported in patients with non-syndromic intellectual disability in 2009 [11]. Since then, an increasing number of cases have been identified with most being caused by truncating mutations, although missense mutations, and microdeletions of *SYNGAP1* have also been described [19, 29, 30]. As of 2018, there had been more than 200 patients reported with *SYNGAP1* mutations [28]. However, there may be many more undocumented individuals with this genetic disorder especially as it is said to account for a high number of cases of sporadic ID in the population.

*SYNGAP1-*related ID has typically been characterised as consisting of moderate to severe intellectual disability, epilepsy, autism, attentional deficits, and/or mood disorders [11, 21, 23]. The prevalence of seizures amongst *SYNGAP1* patients has been reported to be high [14, 19, 21, 27]. For example, Vlaskamp et al. [27] in a cohort of 57 *SYNGAP1* patients found that 98% had epilepsy which were characterised as eyelid myoclonia with absences (65%), myoclonic seizures (34%), atypical (20%), and typical (18%) absences and atonic seizures (14%), which were triggered by eating in 25%. Behavioural problems such as aggression have also been highlighted as characteristic of *SYNGAP1*, with reports ranging from 60% [14] to 73% prevalence in this population [27] whilst autism spectrum disorder (ASD) has been reported in at least half of all patients [3, 14, 19, 21, 27]. Alongside these, Vlaskamp et al. [27] identified other features which were common for *SYNGAP1* patients including high pain threshold (72%), eating problems including oral aversion (68%), hypotonia (67%), sleeping problems (62%), and ataxia/gait abnormalities (51%).

With *SYNGAP1*-related ID only being reported in patients in 2009, there exists only limited research and as a result there is a lack of understanding of the behavioural phenotype of this disorder. To date, the most common method to assess the behavioural aspects of this disorder have relied upon brief questionnaires and identification of existing diagnostic labels. However, this method restricts the respondents’ answers and so insightful information in regard to the behavioural phenotype may be missed, particularly in a relatively newly described condition. To overcome this, in this report we undertake semi-structured interviews with the parents and caregivers of children with *SYNGAP1-*related ID. To our knowledge, this is the first study to undertake this qualitative methodology. This approach will provide a more natural way of interacting with families allowing us to gain richer data and to validate existing research findings. As a result it will help to significantly characterise the clinical and behavioural features of this monogenic disorder. Gaining an increased understanding of the behavioural features of this disorder is important to help inform clinical practise to further aid the identification of additional cases and to help to provide appropriate care to those individuals with *SYNGAP1-*related ID.

## Method

### Participants

Information was obtained from 11 males and 16 females who had all received a diagnosis of *SYNGAP1*-related ID (Table 1). All parents gave informed written consent to provide information about their child, alongside consent for their child’s information to be included in the study. The study protocol was reviewed and approved by NHS Scotland A Research ethics committee. Families were recruited through the Bridge the Gap–SYNGAP Education and Research Foundation and via word of mouth.

**Table 1:** Description of the participants

	Male	Female	Total
**Total**	11	16	27
**Age (mean)**	9.4	7.8	8.4
**Age (SD)**	8.7	5.3	6.8

### Data collection and analysis

The interview consisted of a series of semi-structured questions with the parents/carers of children with *SYNGAP1*-related ID. The questions focused on eight areas: basic information (e.g., age gender, genetic variation), family and perinatal history, past medical history, developmental history, epilepsy, ASD and attention deficit hyperactivity disorder (ADHD) diagnosis, sensory sensitivities, and general behaviour. For each area, there were a set of specific questions that were asked (see Supplementary material). To elicit further information, these questions were then followed up with additional probes and questions. The interview was designed to allow parents to give a broad explanation in their own words of their child’s abilities and behaviour. Interviews were conducted by two of the authors (DW and AK) and one additional interviewer, either face-to-face or remotely (on the phone or via video call). The parents and care givers answers were transcribed and prior to analysis any identifiable information was anonymised.

A mixed methods approach was utilised to examine the responses of the parents/carers. A quantitative approach consisting of reporting descriptive statistics was taken to examine the responses that the parents gave concerning family and perinatal history, past medical history, developmental history, epilepsy and ASD, and ADHD diagnosis. A qualitative inductive approach content analysis [7] was performed on the responses that the parents give about their child’s sensory sensitivities, general behaviour and the three behaviours that they or their child found the most difficult. Responses about sensory sensitivities were analysed separately from parents’ responses about their child’s general behaviour. This approach allowed for a systematic and objective means of describing of these behaviours. Firstly, codes were generated using NVivo (version 12) to identify relevant content and patterns. Researchers then came together to discuss these and categories then emerged. Researchers separately went away to combine these into themes which were then discussed and agreed upon. As DW and AK were involved with data collection to ensure the credibility and robustness of the data interpretation, an external researcher was also involved in data analysis (SE).

To enhance the validity of our findings, we conducted post-analysis data verification. We ensured that saturation had been reached by using the method described by Guest, Namey, and Chen [9] which determined that this had occurred at 14 + 2 interviews (base size of 6 with a ≤ 5% new information saturation threshold). Further, member checking was performed by sending participants a summary of the themes and findings of the interviews in order for them to confirm the accuracy of our interpretations. All subjects who responded to our request agreed that our summary was consistent with the behaviour phenotype that they had observed in their own child.

## Results

### Family, perinatal, and past medical history

All parents reported being non-consanguineous, with 16 parents reporting no previous cases of ID, ASD, ADHD or epilepsy in the first-degree family. Eleven reported that they had extended members of the family (e.g., cousin, uncle, distant relative) who had displayed some form of ID, ASD, or developmental delay.

In regard to perinatal history, all pregnancies proceeded to full-term with the average gestational age being 39 weeks. None of the parents reported a preterm birth prior to 37 weeks. Of the 27 cases, 23 were vaginal births, with five of these being induced, and another three being ventouse deliveries. The remaining four births were via caesarean section. Only one child was reported to have had a very low birth weight (less than 1.5 kg), with the average birth weight being 3.25 kg (min 1.3 kg; max 4.9 kg). Twenty-four parents reported having no exposure to any known teratogens. Those that did reported exposure to flu, listeria infection and the rubella vaccine.

Parents highlighted a range of different conditions that had been experienced by their child including hayfever (4 individuals), reflux (4), dry skin (2), hip dysplasia (2), pneumonia (2), coughs and colds (2), kyphosis (2), and glue ear (2). There were also a number of conditions that were mentioned just once these included febrile convulsion, asthma, ear infections, focal cortical dysplasia, nystagmus, gastrostomy, mycobacteria abscess, hyperinsulinaemia, early menstruation, precocious puberty, depression, anxiety, chest infections, and food-protein induced enterocolitis syndrome. Five parents reported that their child had no previous medical history of note to report.

### Epilepsy

Epileptic seizures were reported in 19 out of 27 of our participants, with the average age of onset being 4 years old. Two parents reported that their child had displayed some seizure-like behaviour (e.g., paroxysmal spasms) but they had not received a diagnosis of epilepsy.

Of those children that had received an epilepsy diagnosis, they experienced a range of different seizure types. The most prevalent were absence, followed by atonic, myoclonic, and tonic seizures, and Lennox-Gastaut syndrome. In order to treat their epileptic seizures they had been prescribed a range of different treatments including Sodium Valproate (10), Clobazam (6), Levetiracetam (5), Lamotrigine (4), Ethosuximide (3), Cannabidiol (CBD) oil (1), and the Ketogenic diet (1). Two families who had received a diagnosis of epilepsy reported that their child was currently unmedicated for their seizures.

### Developmental history

Developmental history examined language development along with fine and gross motor skills.

In terms of normal development, it would be expected that fine motor skills, particular grasping ability would be developed between 6 and 12 months of age, whilst for gross motor skills by 19–24 months most toddlers would be able to walk unassisted. For language, first words would be produced by the age of 2. However, almost all parents reported that their *SYNGAP1* child had some form of developmental difficulty. Fine motor skills varied amongst respondents with five using a fist grip and fourteen using a pincer grip to pick up objects.

For gross motor skills, 21 parents reported that their child was able to walk. However, 12 parents stated that they were unsteady and would stumble when performing this action, which may be the result of ataxia and gait abnormalities which were present for 17 children. Of the children who could walk, six reported that they were also capable of running. All of our samples were aged over three years of age and so these skills (walking, running, and picking up objects) were expected to be present in a typically developed age equivalent cohort. Other gross motor deficits highlighted included low muscle tone (9), hypermobility (3), and dyspraxia/poor coordination (14).

Language abilities were mixed with twelve children reported as being nonverbal (only used gestures, facial expressions, and eye contact to express themselves), whilst seven had better language skills and so were able to articulate either single or multiple words. Of those that were nonverbal or only able to express single words, nine were still able to demonstrate understanding and were able to express what they wanted either through signing or via communicating physically.

### Behavioural phenotype

#### Diagnoses

In total, 52% had received a diagnosis of ASD whilst 7% reported having received a diagnosis of ADHD. One child had received a diagnosis of both ASD and ADHD.

#### General description

**Fig. 1:**
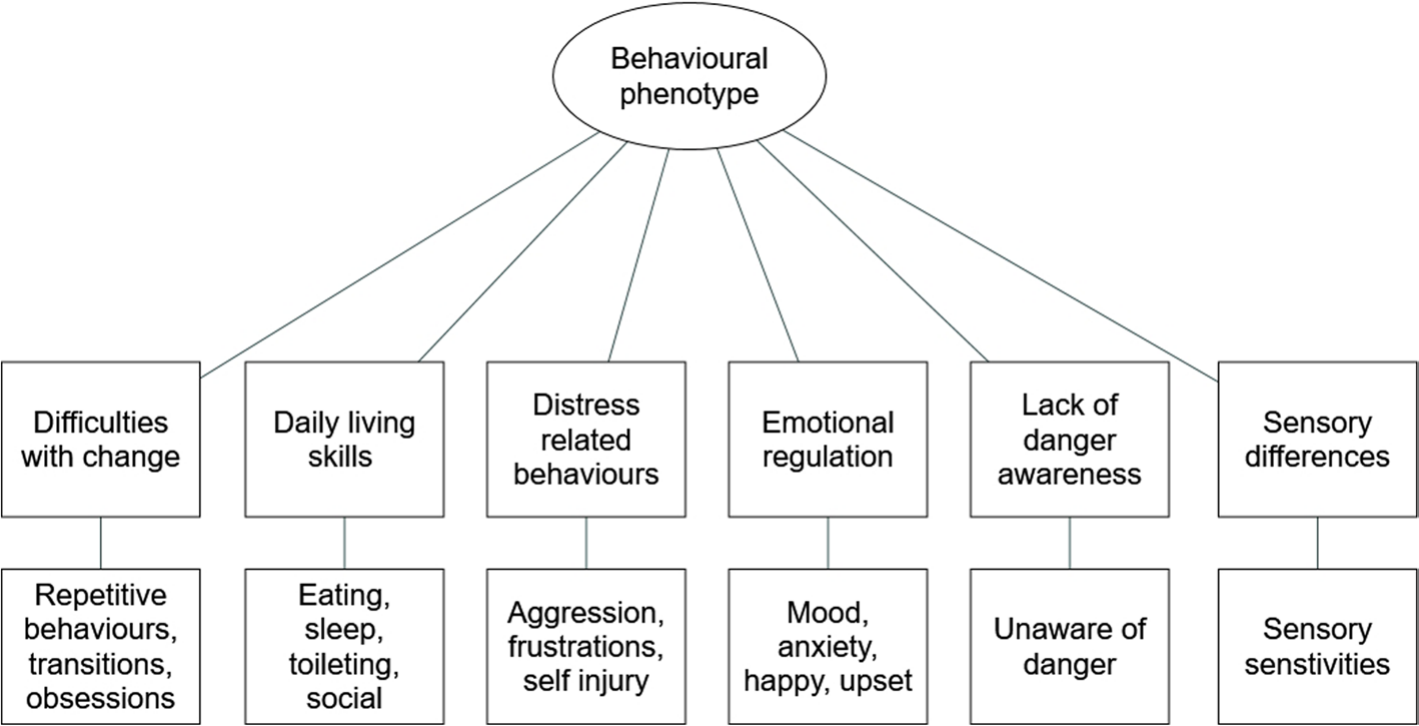
Themes and sub-themes of the behavioural phenotype of *SYNGAP1*-related ID

Parents were separately asked to give a general description of their child’s behaviour and then to outline the three main behaviours that caused them or their child the most difficulty. The behavioural phenotype of *SYNGAP1* was found to relate to themes of daily living skills, distress-related behaviours, emotional regulation, difficulties with change, a lack of danger awareness, and sensory differences (Fig. 1).

##### Daily living skills

A common theme that emerged was difficulties with daily living skills. These quite often concerned eating, sleeping, toileting and social. Sleep was an issue that was often reported by the parents/carers. These sleep issues concerned not only the bedtime routine which for some would take an extended period, but parents reported also that their children would wake repeatedly during the night: ‘Sleep is a huge problem. Very irregular and needs to stick to extremely strict bedtime routine, which takes about an hour and a half’. Another parent commented ‘Sleep is a problem—isn’t on melatonin or anything and usually okay to fall asleep—but wakes during the night and can be wide awake.’ Quite often sleep patterns of *SYNGAP1* children were reported to be irregular: ‘Sleep patterns comes and goes in waves—worse when she was baby and still naps in afternoon’. However, some reported that medication seemed to help to ease these difficulties with sleeping: ‘Sleep is better since being on melatonin but will wake around 3/4am and may go back to sleep or be awake for the rest of the night’.

Despite having no or limited language abilities many of the children were still reported to be receptive and keen to have social interactions with others. For example: ‘Enjoys other children. Follows other children in active activities. Will approach other children’. However, this social interaction could sometimes be selective to certain individuals: ‘Has some social interaction with a couple of children in his class. Will ask for one particular child if they are absent and initiates interaction with them’ and ‘he communicates and plays well with his sister. Doesn’t play with others well due to a lack of understanding’ whilst others commented that their child ‘prefers older children and adults’. However, this interest was not universal with other parents highlighting that their child was shy, uninterested in interactions with others or lacking in social skills. Further, many parents reported that their child had good eye contact and would often use it to get attention.

Toileting and eating were two other daily living skills that parents commented on. Some reported that their child had no response to toileting or they had difficulty in going: ‘No sensory response for toileting’ and ‘knows how to go but sometimes doesn’t’ whilst some highlighted that constipation was a problem. Feeding was another issue mentioned by parents. For example, one commented that their child was ‘not a great eater—will try everything but just doesn’t eat a lot. Also has a very sweet tooth’.

##### Distress-related behaviours

Behaviours consisting of frustration and aggression were a common theme mentioned by the majority of parents. The behaviours demonstrated by those with *SYNGAP1* involved being frustrated and aggressive and parents noted this was often due to them being denied something, unable to get their own way or unable to understand the situation. For example, one parent commented that their child: ‘gets anger meltdowns if he doesn’t get his own way’. Quite often this frustration and aggression would result in violence towards other individuals but also towards themselves as illustrated by some parents comments that included ‘tends to swipe at other children and adults in an aggressive way’ and ‘bites herself when she gets frustrated and can scratch others’. This self-injurious behaviour often consisted of them biting their hands, head-banging, and face-hitting.

##### Emotional regulation

When the parents were prompted about the general mood of their child, they often expressed that they were happy; however, this could change and vary quite quickly to them being upset and frustrated: ‘Gets very upset if fails at something’ and ‘changes from happy to upset quickly’. Anxiety was also mentioned as a concern by a number of parents.

##### Difficulties with change

Many of the parents highlighted that they were also prone to issues involving transitions and repetitive behaviours. The repetitive behaviours involved various different stimuli or actions, for example one parent commented that their child had ‘many repetitive behaviours like walking up and down stairs and switching on and off lights’, whilst others highlighted repetitive behaviour to sounds, play and the repeating of phrases. Transitions and changes in routines were also issues for the *SYNGAP1* individuals: ‘Doesn’t like change from routine’, ‘doesn’t understand now and next’, ‘has around 5-10 outbursts a day, often related to transitions or having to do something she doesn't want to’ and ‘difficult when there is a change in routine’. One parent reported that if the change in routine was small then their child was able to cope with this, however they had issues when it came to transitions: ‘copes well with small changes if well managed but doesn't like transitions’. Also, one parent reported that their child had shown signs of regression with their interests: ‘Has regressed to toddler TV and gets angry and upset with TV for older children. Obsessed with shopping trolleys and fixated on routine’.

##### Lack of danger awareness

Some of the parents described their child as lacking an awareness of potential dangers in the world. In particular, one parent commented that their child would try to pull away from the parent when out in the community: ‘Lack of danger awareness and bolting behaviour – will try to pull away and escape when out and about and has escaped from buildings’.

##### Sensory differences

This theme emerged as parents described difficulties stemming from sensory sensitivities such as ‘bothered by loud noises’ and ‘obsessed with gloves’. Some highlighted that their child had strong sensory responses with comments including ‘requires very high stimulation’ and ‘can get overstimulated easily’. Parents brought these issues up spontaneously when discussing behavior, but sensory differences were also explored in more detail through a specific enquiry (see below).

#### Sensory profile

**Fig. 2:**
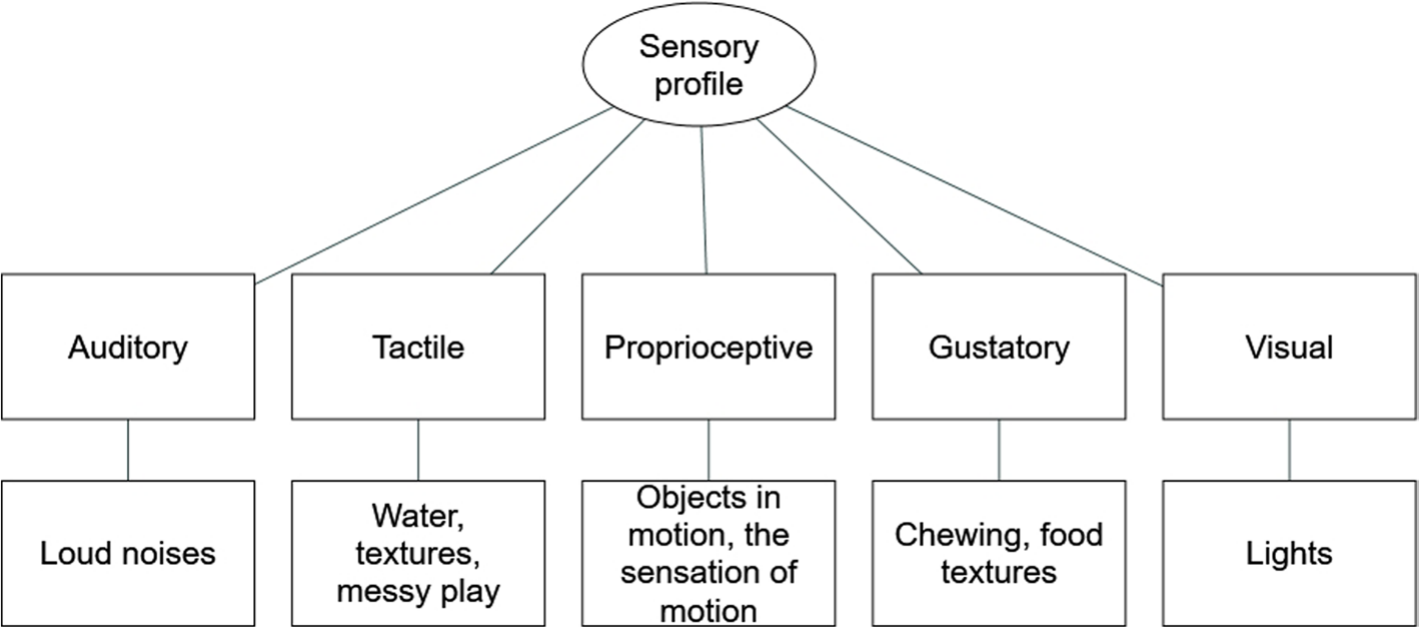
Themes and sub-themes of the sensory profile of *SYNGAP1*-related ID

Many parents and carers reported that their child seemed to respond either particularly positively or negatively to sensory stimuli. In some cases there were specific sensory sensitivities, whereas in other cases parents and caregivers noted that their child seemed to particularly like or be soothed by particular sensory stimuli. These centered on visual, tactile, proprioceptive, gustatory, and auditory modalities (Fig. 2).

##### Tactile

Parents highlighted that many children responded particularly to textures, touch, and messy play. In particular, water was frequently reported to be a texture that the children were fixated with and found to be pleasurable to experience: ‘loves running water and bubbles—used to have to cover the sinks at nursery—has to stay in bath until water is drained and if shower is running can get into a tantrum if try to turn it off’. It was also mentioned that textures could be sensations that the children found quite pleasurable whilst others found them aversive. For example, one parent highlighted that their child liked a range of textures: ‘Loves water/swimming, slime and shaving foam’. However, other parents said that textures were sensations that were not particularly liked: ‘dislikes textures (e.g., sand/paint/wet things/soft/sticky)’, whilst messy play was an activity that was particularly disliked by those with *SYNGAP1*: ‘doesn’t particularly enjoy messy foods or messy play’ and ‘hates being dirty or anything messy’.

##### Auditory

Parents often reported that their child had sensitivities to auditory stimuli. This sensitivity was predominantly to loud noises and was particularly aversive to those individuals with *SYNGAP1*. This was emphasised by comments such as ‘hates tannoys and loud speakers’, ‘doesn’t like fire alarms and sounds of people walking’ and ‘hates loud noises and wears ear defenders’.

##### Visual

Some parents highlighted that their child had visual sensitivities such as to bright lights, with these being a stimulus that they either liked or showed no particular aversive behaviour towards: ‘ok with lights/visual’ and ‘loves bright lights’.

##### Gustatory

The parents also highlighted that their child with *SYNGAP1* exhibited sensitives to particular foods and the act of eating. It was commented that some children had a desire to excessively chew and grind their teeth whilst others disliked food textures: ‘doesn’t like food textures’ and ‘orally sensitive and has need to chew, bite, and grind teeth’.

##### Proprioceptive

Another sensory sensitivity that was often commented on by parents/carers concerned the environment and in particular experiences that involved motion. For example, it was often mentioned that the children loved watching objects in motion, or that they enjoyed activities such as being in car or being on a swing which involved the sensation of motion. Comments from parents included: ‘Loves rolling things on floor, dropping things, watching cars and being in cars’ and ‘loves motion and wants to have some sort of movement all the time, loves the trampoline and swing and would stay on them all day’.

## Discussion

*SYNGAP1-*related ID is a relatively recently documented neurodevelopmental disorder, and as a result reports outlining its behavioural phenotype are somewhat lacking. Those that have highlighted the behavioural phenotype of *SYNGAP1-*related ID have previously been in the form of questionnaires, EEGs and clinical examinations. To our knowledge, this is the first study to undertake semi-structured interviews with the parents and care givers of children with *SYNGAP1-*related ID. This approach allowed for specific topics to be explored whilst allowing opportunities for open ended responses in order to provide a greater understanding of the behaviour phenotype of this disorder. As a result, this study adds significantly to our limited understanding of the behavioural features of *SYNGAP1*.

In keeping with the existing literature, our quantitative analysis of the caregiver responses found that developmental delays, epilepsy, and ASD were particularly common in those with *SYNGAP1*. Developmental deficits were prevalent across our sample with many parents reporting limited fine and gross motor skills alongside language impairments. Fine motor skills consisted of limited abilities to pick up objects using either a pincer or a fist grip, whilst for gross motor skills many could walk but were reported to have ataxia and dyspraxia. This is consistent with previous reports which have highlighted that ataxia and gait abnormalities are a frequent feature of *SYNGAP1-*related ID occurring in up to 51% of cases[19, 27]. Language was also demonstrated to be impaired with nearly half of the participants reported as being non-verbal, whilst those with greater language abilities had only progressed to single word or simple sentences. Mignot et al. [19] and Vlaskamp et al. [27] also reported similar language abilities in their cohorts. This data suggests that children with *SYNGAP1-*related ID initially made some developmental progress but this soon levels off resulting in limited motor and language abilities.

In regard to the prevalence of epilepsy and seizures, 70% of our cohort reported a diagnosis of epilepsy whilst we had a further three patients that had suspected epilepsy but had not received a diagnosis. This finding is lower than has been found previously, with epilepsy reported as present in around 98% in the *SYNGAP1* samples formerly described [19, 27]. This may relate to differences in sample ascertainment between studies—we recruited primarily through a support organisation, whereas other samples have been derived from clinical practices [27] or cohorts with individuals investigated for epilepsy [19] where the prevalence of seizures would be expected to be higher. Meanwhile, a diagnosis of ASD had previously been reported to be a feature for between 50% and 54% of patients with *SYNGAP1-*related ID [19, 27]. Our study was consistent with this, with 52% of our sample having received a diagnosis of ASD.

To allow for a complete picture of the behavioural phenotype of *SYNGAP1*, we also asked the caregivers about the general behaviour of their child. The themes that emerged concerned daily living skills, distressed-related behaviours, emotional regulation, difficulties with change, a lack of danger awareness, and sensory dysfunction. Many of these behaviours concerned activities that their child had issues with and as such further emphasises the significant impact across multiple domains that *SYNGAP1* can have on an individual’s life. Further, many of these themes were in line with previous quantitative research which have suggested that the behavioural features of *SYNGAP1* are characterised by aggression and sensory issues [19, 23].

Our qualitative analysis of the parents’ responses highlighted that the sensory sensitivities of those with *SYNGAP1* centered on audio, visual, tactile, proprioceptive, and gustatory themes. It is noted that many of our participants have a diagnosis of autism and future research could aim to clarify how these sensitivities relate to other autistic traits in this population. Previously, Michaelson et al. [18] had reported that *SYNGAP1* patients had reported sensory abnormalities being prevalent with an emphasis of these being on responses to tactile stimuli. Indeed, tactile issues appear to be a common phenotype of *SYNGAP1*, with *syngap1* mouse models also exhibiting these abnormalities [6, 18]. Our study has been able to not only further outline these behaviours to specific textures and tactile stimuli such as water and messy play but to also highlight other stimuli that those with *SYNGAP1* find particularly pleasurable or aversive; for example motion and loud noises respectively.

Together, these findings illustrate that those with *SYNGAP1* exhibit a range of behaviour difficulties, including sensory sensitivities, language difficulties, and repetitive behaviours; with many of these consistent with behaviours identified in other IDD conditions as well as in ASD. Sensory processing is altered in a number of neurodevelopmental genetic disorders including ID and ASD [15]. Behavioural and electrophysiological studies have demonstrated sensitivities and alterations in sensory processing, particularly in the auditory domain for both ASD [15] and Fragile x syndrome (FXS [4, 24];), which is a leading cause of intellectual disability and ASD. Repetitive and restricted behaviours are also commonly observed in both FXS [20] and ASD [5], whilst delays in speech/language and gestural development [1, 13] have also been demonstrated. As such this study provides further evidence of the behaviour difficulties experienced by those with intellectual disabilities and ASD. The highlighting of these behavioural characteristics should help to provide focus to interventions and further establish a core diagnostic criteria for *SYNGAP1*-related ID.

However, the findings of our study should be interpreted in the context of some limitations. The study relied on interviews in order to describe *SYNGAP1*-related ID phenotype. Whilst this approach allows parents the opportunity to describe their child’s behaviour in their own words, leading to a greater flexibility in response than may be found with structured questionnaires, it has some drawbacks. First, due to the interviews being semi-structured and the answers allowed to be open-ended, it meant that the information we obtained may sometimes have been incomplete and was not standardised between respondents. Second, the responses may have been biased by some parents to highlight specific features of their own child’s behaviour whilst neglecting others that they felt were less important. These limitations may have limited the interpretations that were made and so may not completely reflect all of the behavioural phenotypes of *SYNGAP1*-related ID. As a result, further studies are required to provide additional descriptions of the characteristics associated with *SYNGAP1*-related ID. For example, in future it may be important to examine the phenotype relating to specific developmental stages and how the features of this disorder manifest with the progression of age.

## Conclusions

In conclusion, this study has set out the behavioural phenotype of *SYNGAP1*-related ID as described by the parents and care givers of children with this disorder. It has highlighted that *SYNGAP1*-related ID is characterised by a high prevalence of epilepsy, autistic behaviours, sensory sensitivities, and developmental difficulties.

## Supplementary Information

**Additional file 1.** Supplementary material. Interview guide.

## Data Availability

The data that support the findings of this study are available from the corresponding author upon reasonable request.
