# Supplementary material for: Clinical and behavioural features of SYNGAP1-related intellectual disability: a parent and caregiver description
Source: J Neurodev Disord. 2022 Jun 2;14:34. doi: 10.1186/s11689-022-09437-x (PMC9164368; doi:10.1186/s11689-022-09437-x)
Supplement: Supplementary file 1 — Additional file 1. Supplementary material. Interview guide. [file 11689_2022_9437_MOESM1_ESM.docx]

**I – Basic information**

| Date: |  |
| --- | --- |
| Interviewer: |  |
| Patient ID: |  |
| Age: |  |
| Gender: |  |
| Genetic Variant: |  |
| Origin of the mutation (*de novo* vs inherited): |  |

**II - Family history**

| Consanguinity (yes/no): |  |
| --- | --- |
| History of learning difficulties, epilepsy, autism or ADHD in first-degree relatives:  Draw a three generation pedigree with details |  |

**III - Perinatal history**

| Exposure to potential teratogens: |  |
| --- | --- |
| Mode of delivery: |  |
| Gestational age at delivery: |  |
| Complications during or post- delivery: |  |
| Birth weight: |  |

**IV – Past medical history (including medication)**

|  |
| --- |

**V - Developmental history**

| Gross motor development  (detailed description): |  |
| --- | --- |
| Fine motor development  (detailed description): |  |
| Language development (detailed description): |  |

**VI - Epilepsy**

| Yes/no: |  |
| --- | --- |
| Age at onset: |  |
| Description of seizures:  (type, frequency, length) |  |
| Description of treatment and response to treatment: |  |
| Other neurological problems: |  |

**VII – Behavioral history**

| Autism spectrum disorder (yes/no): |  |
| --- | --- |
| ADHD (yes/no) |  |
| Sensory sensitivities |  |
| Description of behavior:  **General description**  e.g.  anxiety  agitation  aggression  eye contact  social interaction  stereotyped behavior  resistance to change  **What are the three main behaviours that cause you or your child difficulty?** |  |
